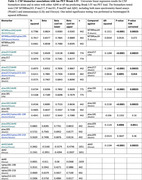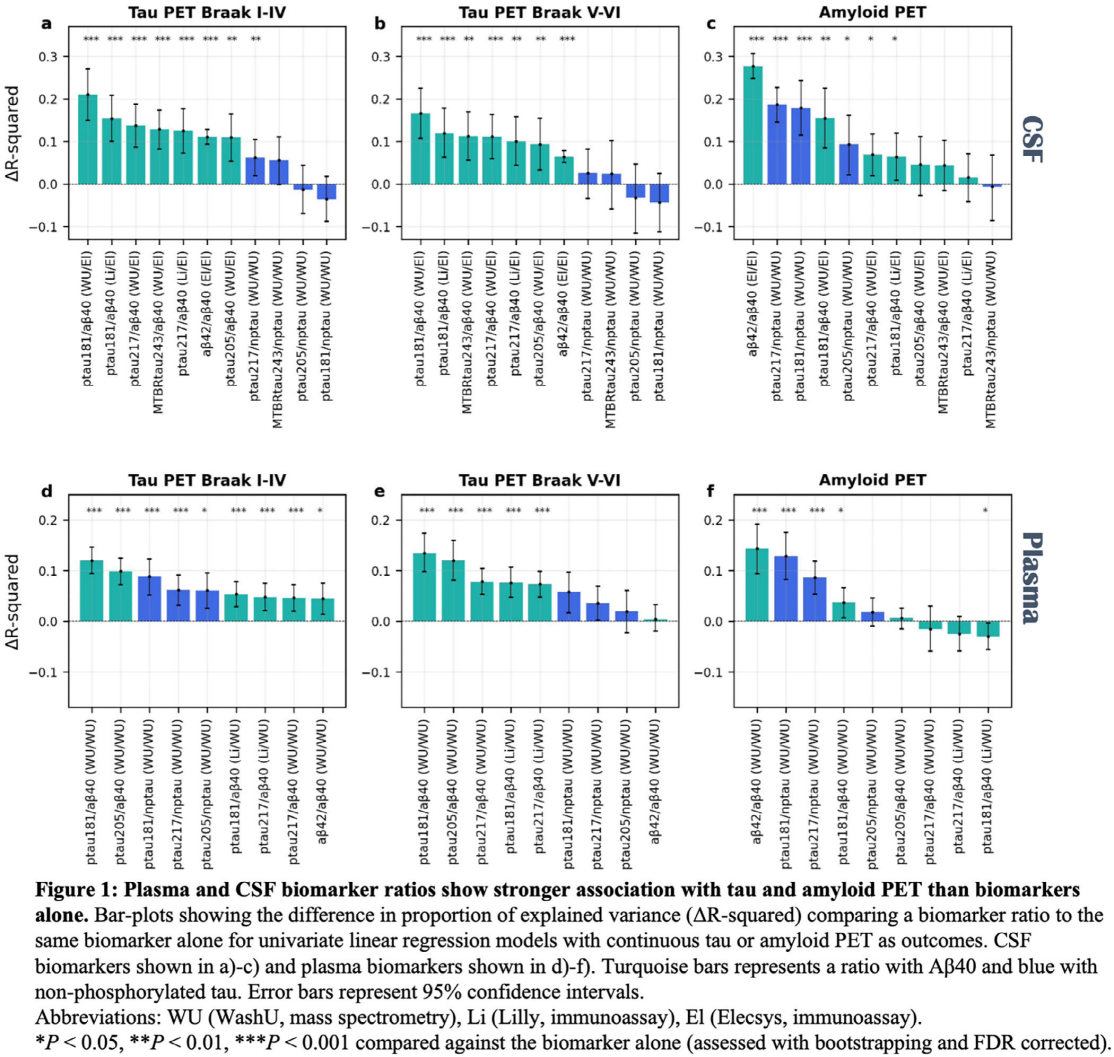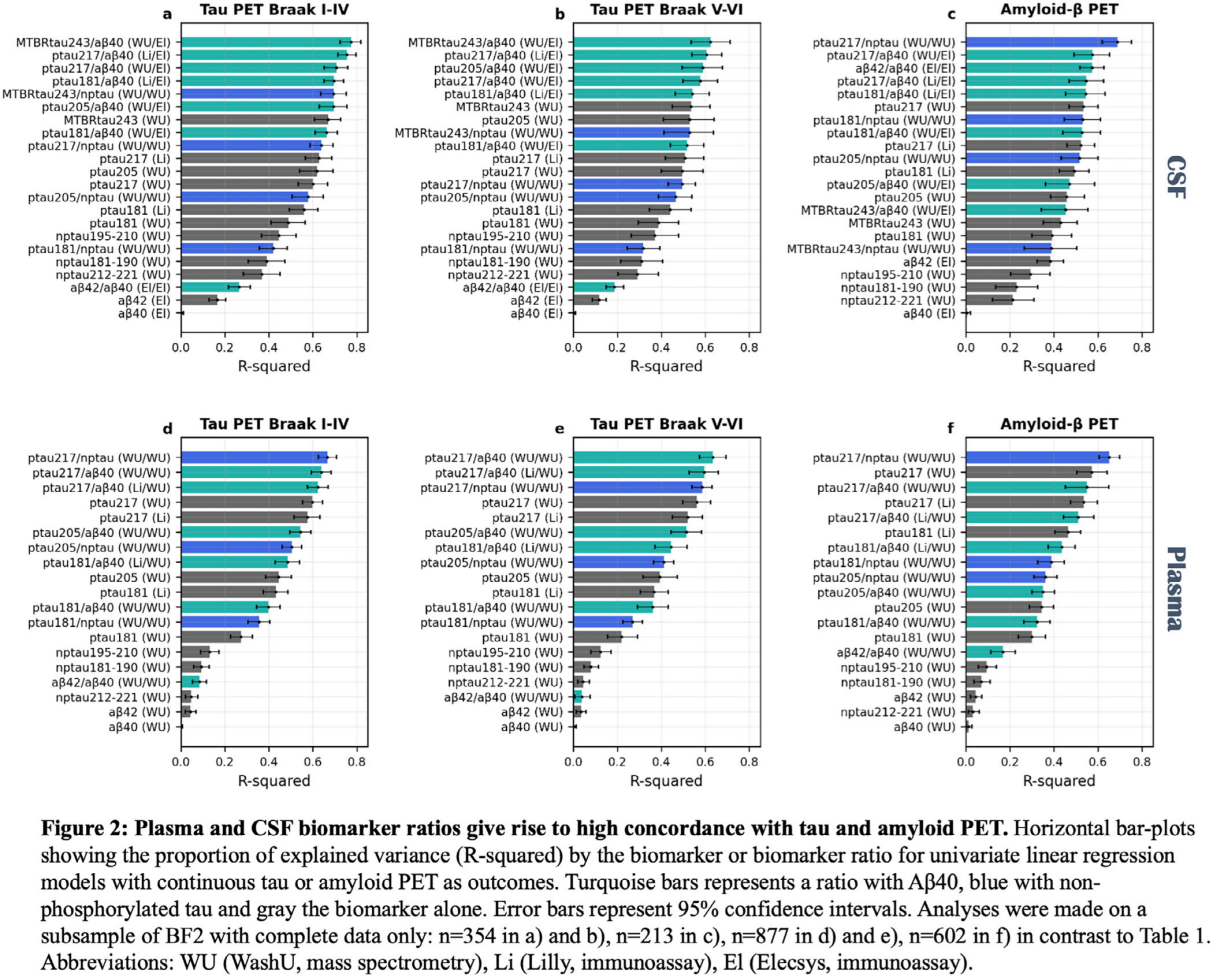# Improved precision of Alzheimer’s disease fluid biomarkers when using Amyloid‐β_40_ or non‐phosphorylated tau as a reference

**DOI:** 10.1002/alz.085036

**Published:** 2025-01-09

**Authors:** Linda Karlsson, Shorena Janelidze, Kanta Horie, Nicolas R. Barthélemy, Jacob W. Vogel, Ida Arvidsson, Kalle Åström, Vitaliy Ovod, James G. Bollinger, Yingxin He, Sebastian Palmqvist, Erik Stomrud, Gemma Salvadó, Alexa Pichet Binette, Kaj Blennow, Randall J. Bateman, Niklas Mattsson‐Carlgren, Oskar Hansson

**Affiliations:** ^1^ Clinical Memory Research Unit, Lund University, Lund Sweden; ^2^ The Tracy Family SILQ Center, St. Louis, MO USA; ^3^ Department of Clinical Sciences Malmö, SciLifeLab, Lund University, Lund Sweden; ^4^ Centre for Mathematical Sciences, Lund University, Lund Sweden; ^5^ Memory Clinic, Skåne University Hospital, Malmö Sweden; ^6^ Department of Psychiatry and Neurochemistry, Institute of Neuroscience and Physiology, The Sahlgrenska Academy, University of Gothenburg, Mölndal Sweden; ^7^ Clinical Neurochemistry Laboratory, Sahlgrenska University Hospital, Mölndal Sweden; ^8^ The Tracy Family SILQ Center, Washington University School of Medicine, St. Louis, MO USA; ^9^ Department of Neurology, Skåne University Hospital, Lund Sweden; ^10^ Wallenberg Center for Molecular Medicine, Lund University, Lund Sweden

## Abstract

**Background:**

Fluid biomarkers represent an informative and cost‐effective way to detect and monitor Alzheimer’s disease (AD). However, as we recently showed, the overall proteome average in CSF exhibits a non‐disease related average signal (inter‐individual variability), which can reduce the precision of concentration based CSF AD biomarkers.^1^ Now, we therefore investigate if several already high performing CSF and plasma AD biomarkers can be improved by normalizing their concentration to a reference protein (e.g. Aβ40 and non‐phosphorylated mid‐region tau [nP‐tau]).

**Methods:**

Using the BioFINDER‐2 (BF2) cohort (n=1908), we compared the associations between amyloid/tau‐PET load and CSF biomarkers (MTBR‐tau243, P‐tau217, P‐tau181, P‐tau205, Aβ42) alone versus in a ratio with a reference protein (e.g. CSF Aβ40 or nP‐tau) in linear regression models. We repeated this analysis for plasma biomarkers (P‐tau217, P‐tau181, P‐tau205, Aβ42) alone versus in a ratio with plasma Aβ40 or nP‐tau. Assays included mass spectrometry based (WashU) and immunoassays (Lilly and Elecsys).

**Results:**

All concentration based CSF biomarkers, including MTBR‐tau243, showed significantly stronger association with tau‐PET load when applied in a ratio with CSF Aβ40 compared to alone (ΔR‐squared=0.0639‐0.213), Table 1 and Figure 1a‐1b. Similar results (but not all significant) were seen for associations with amyloid‐PET (ΔR‐squared=0.0161‐0.278), Figure 1c. A ratio with CSF nP‐tau significantly increased biomarker concordance with amyloid‐PET for all except MTBR‐tau243 (ΔR‐squared=‐0.0059‐0.188), but not tau‐PET (ΔR‐squared=‐0.044‐0.0626). For plasma biomarkers, ratios with both Aβ40 and nP‐tau led to stronger associations with tau‐PET (ΔR‐squared=0.0039‐0.120), while associations with amyloid‐PET mainly were improved in ratios with nP‐tau (ΔR‐squared=0.018‐0.123), Figure 1d‐1f. When comparing all biomarkers (Figure 2), highest correlation with tau PET was seen for MTBR‐tau243/Aβ40 in CSF (R‐squared=0.770) and P‐tau217/nP‐tau in plasma (R‐squared=0.666), and with amyloid‐PET for P‐tau217/nP‐tau in both CSF (R‐squared=0.689) and plasma (R‐squared=0.652).

**Conclusions:**

CSF and plasma biomarkers show stronger association with tau‐PET and amyloid‐PET loads when applied in a ratio with a reference protein (e.g. Aβ40 or nP‐tau). Ongoing work involves validation in external cohorts, and longitudinal analyses to assess if a ratio approach also can reduce biomarker fluctuations within an individual.

Karlsson, L. et al. Cerebrospinal fluid reference proteins increase accuracy and interpretability of biomarkers for brain diseases. BioRxiv (preprint) (2023) doi:10.1101/2023.06.08.544222.